# Evaluation of data accuracies within a comprehensive geospatial-health data surveillance platform: SOMAARTH Demographic Development and Environmental Surveillance Site, Palwal, Haryana, India

**DOI:** 10.1017/gheg.2018.17

**Published:** 2018-12-27

**Authors:** Natasha J. Howard, Shikha Dixit, Hasan Raja Naqvi, Atiqur Rahman, Catherine Paquet, Mark Daniel, Narendra K. Arora

**Affiliations:** 1 Wardliparingga Aboriginal Research Unit, Division of Health Sciences, University of South Australia, North Terrace, Adelaide, South Australia, Australia; 2 South Australian Health and Medical Research Institute, Adelaide, South Australia; 3 SOMAARTH Demographic Development and Environmental Surveillance Site, International Clinical Epidemiology Network (INCLEN) Trust, New Delhi, India; 4 Department of Geography, Faculty of Natural Sciences, Jamia Millia Islamia, New Delhi, India; 5 School of Health Sciences, University of South Australia Division of Health Sciences, Adelaide, South Australia, Australia; 6 Health Research Institute, University of Canberra Faculty of Health, Canberra, Australian Capital Territory, Australia; 7 Department of Medicine, St. Vincentʼs Hospital, The University of Melbourne, Melbourne, Australia

**Keywords:** geographic information system, global health, other, position and attribute accuracy, spatial data accuracy, surveillance and monitoring

## Abstract

Evidence exists of an increasing prevalence of chronic conditions within developed and developing nations, notably for priority population groups. The need for the collection of geospatial data to monitor the health impact of rapid social-environmental and economic changes occurring in these countries is being increasingly recognized. Rigorous accuracy assessment of such geospatial data is required to enable error estimation, and ultimately, data utility for exploring population health. This research outlines findings from a field-based evaluation exercise of the SOMAARTH DDESS geospatial-health platform. Participatory-based mixed methods have been employed within Palwal-India to capture villager perspectives on built infrastructure across 51 villages. This study, conducted in 2013, included an assessment of data element position and attribute accuracy undertaken in six villages, documenting mapping errors and land parcel changes. Descriptive analyses of 5.1% (*n* = 455) of land parcels highlighted some discrepancies in position (6.4%) and attribute (4.2%) accuracy, and land parcel changes (17.4%). Furthermore, the evaluation led to a refinement of the existing geospatial health platform incorporating ground-truthed reflections from the participatory field exercise. The evaluation of geospatial data accuracies contributes to understandings on global public health surveillance systems, outlining the need to systematically consider assessment of environmental features in relation to lifestyle-related diseases.

## Background

Increased attention has been drawn to the role of environmental factors in the aetiology of chronic diseases, such as cardiovascular disease and type 2 diabetes [1–3]. Notably, there is a need to focus on the burden of contemporary population health challenges experienced within low- and middle-income countries [4]. Mohindra *et al.* [5] have specifically outlined for India the ‘high level of health needs and new public health challenges arising in the context of rapid economic growth and social change (p. 1)’. Key strategies and methods recommended for pursuing an equity-oriented public health research agenda were the investment in data systems and development of inter-disciplinary approaches. Despite the importance of these recommendations, there has been limited platforms focusing on the need for collection, maintenance and surveillance of geospatial-health data systems to address complex social determinants of health embedded within the places in which people live [6–8].

Traditionally, public health surveillance systems have been defined by the World Health Organization (WHO) as ‘the continuous, systematic collection, analysis and interpretation of health-related data needed for the planning, implementation, and evaluation of public health practice’ [9]. Broadening public health surveillance systems to incorporate geospatial information on risk conditions beyond conventional health-related data will require in the first instance the systematic capture of detailed environmental influences on health. Demographic and health surveillance systems within low- and middle-income countries, such as those aligned with the INDEPTH network [10], have emerged to explore the dynamic nature of population health, within and across communities. An expansion beyond traditional health issues, such as infectious diseases, vaccine sciences, reproductive health and access to safe water, have been supported by calls for surveillance systems to explore behavioural risk factors associated with chronic diseases [11]. Moreover, the rapid social and economic changes also require systems to dynamically capture demographic and health changes in relation to the environmental risk conditions, through the application of remote-sensing and geographic information system (GIS) technologies. To date, demographic and health surveillance systems within low- and middle-income countries have not routinely captured environmental risk conditions. Evidence on which risk conditions to intervene on is poor, however, and exacerbated by a lack of data infrastructures that integrate the environmental (i.e. spatial) exposures, risk factors and health outcomes needed to elucidate the environmental factors and mechanisms as applied across or within different settings. Most notably, advances in geospatial-health infrastructures will be required within disadvantaged and minority populations.

Matthews *et al.* [12] have supported the need for the collection of geospatial data, yet concurrently have outlined the vital requirement to assess these data for quality, and thus utility for application within population health. Research into place and health has seen the undertaking of geospatial data validation of existing administrative data sources such as seen with food retail and physical activity resources [13], and valuation or landuse databases [14], for example, commercial database validation using the Australian telephone directory, ‘Yellow Pages’ [15]. Such research confirms that information obtained from commercial databases must be treated with caution; nonetheless, these sources have potential to further our understandings on place–health relations. Zhang *et al.* have outlined the importance of understanding measurement errors for applications in spatial epidemiology, yet did not explore beyond the inaccuracies of geospatial reference points (e.g. *x* and *y* coordinates in space) [16]. Furthermore, assessments of applications such as Google Street View [17] and Google Earth [18] have been undertaken. Other assessments of geospatial data have seen the collection within urban areas of residential features according to a direct observation scale [19]. Field-based measurement tools are beneficial in their ease to administrate and ability to capture aspects of the built and social environment, as well as demonstrating value within rural regions where access to secondary data sources is limited. There has been no study to our knowledge within low- and middle-income countries that has undertaken a validation of environmental data features integrated with social or health surveillance systems.

To address the identified need for geospatial health surveillance platforms, the International Clinical Epidemiology Network (INCLEN) Trust developed a large-scale surveillance system within the Palwal District, Haryana, India – the SOMAARTH†^1^ Demographic Development and Environment Surveillance Site (DDESS) [20]. During the SOMAARTH platform preparation, a field-based evaluation of geospatial data element accuracy was undertaken within rural Indian villages undergoing rapid environmental, economic and social change. The encounters during the evaluation informed the refinement of data elements and will provide future directions and considerations for public health surveillance data systems within the context of low- and middle-income countries.

## Methods

### Study background

**Fig. 1.**
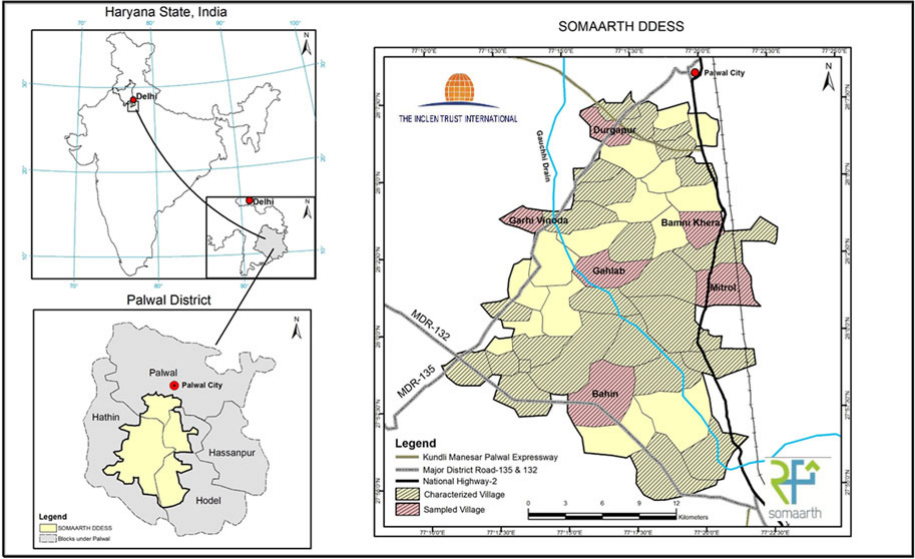
SOMAARTH Demographic and Development Environmental Surveillance Site, study region, Palwal District, Haryana State, India.

SOMAARTH DDESS is located in Palwal District, the 21st District of Haryana State, covering a regional area of 135 933 hectares and divided into four blocks; Palwal, Hodal, Hassanpur and Hathin (Fig. 1).

The Palwal District relies predominantly on an agricultural industry, with these foundations crucial to the region's economic livelihood. There are rapid social, economic and urban form changes being witnessed and anticipated in the near future with the development, in 2011, of the Kundali-Manesar-Palwal (KMP) Expressway. Furthermore, the villages that are located in proximity to the National Highway-2 (NH-2) Delhi-Agra Highway and Delhi National Capital Region (NCR) are experiencing transformation of agrarian land to educational, commercial and industrial lands, most notably with new manufacturing and service industries within these areas. These developments most likely reflect the population growth of 25.5% between 2001 and 2011 in the Palwal District [21].

### SOMAARTH DDESS geospatial health platform

The SOMAARTH DDESS includes 51 villages and three blocks which are bounded by the NH-2 on the east, Palwal-Mewat State Highway on the north-western side and Nuh-Hodal State Highway on the south side (Fig. 1). The data platform enables measurement of rapid regional development through the village-specific capture of proximities to foci including businesses, industrial and economic zones, road networks, and the nature of the social environment (e.g. education, income, caste and religion). The SOMAARTH DDESS is innovative in the development of a comprehensive GIS that will complement a demographic, development and environmental surveillance system incorporating social, behavioural and health data, allowing for place–health insights into both communicable and non-communicable disease outcomes. Human Research Ethics Committee approvals have been received from the INCLEN Trust International Committee (Ref No.s IIEC 010 and IIEC002) and Lucknow Ethics Committee (Ref 23/LEC/10).

A mixed-method approach to SOMAARTH DDESS geospatial data development included four steps: (1) an on-site participatory exercise with local village members to produce paper-based village maps; (2) digitization of land parcels and features of the built and physical environment using very high-resolution satellite imagery; (3) further attribution, validation and refinement of geospatial data layers through villager and field worker consultation and completion of a population Census and (4) data update and surveillance system maintenance.

QuickBird™ satellite imagery was used to prepare various geospatial data on rural areas and store the information (digital village layer) within a GIS domain [20]. These layers included detail on features at their finest spatial unit; water bodies were represented as polygons, roads as either polygon or line, railway tracts as line features, landmarks as a point location (e.g. tube wells) and dwelling units or non-residential features as a *land parcel*. A land parcel was spatially depicted as a polygon feature representing an area of private or commercial ownership.

Each land parcel was subsequently assigned by the geospatial technician a landuse categorization according to an adapted system [20]. The resulting landuse classification system included three levels. *Level I* representing ‘Built-Up Land’, ‘Agricultural Land’, ‘Water Bodies’, ‘Waste Land’ and ‘Vacant Land’. Built-Up (Level I) was further refined to *Level II*, to include the classifications of ‘Residential’, ‘Commercial’, ‘Industrial’, ‘Institutional’, ‘Utilities and Services’ and ‘Agricultural and Others’. Subsequently, each Level II category had been further refined into a *Level III* classification. The attribute table within the GIS village layer included all land parcels that were characterized within the village. Further information on methodology of SOMAARTH DDESS planning and implementation has been published elsewhere [20].

### Evaluation exercise of SOMAARTH DDESS

Following the SOMAARTH DDESS data creation, the objective of this study was to evaluate position and attribute accuracy for the existing geospatial elements (land parcel, road and landuse classifications) integrated into the geospatial health platform.

Erickson and Baker [22] argue for the importance of assessing the ‘what’ as well as the ‘where’ with regards to the accuracy of data contained within a geospatial data system. Position accuracy entails an assessment of the location (or the ‘where’), for example, through observation of land parcels (e.g. size and shape). Attribute accuracy (or the ‘what’) has been described by Goodchild [23] to be one of the major contributors to the quality of geospatial data, and an attribute can be defined as ‘a fact about some location, set of locations, or feature on the surface of the earth (p. 59)’. Knowledge gained on the levels of error and ‘fitness for the user's need’ through assessing geospatial data element position and attribute accuracy [22] will further interpretations of the relationship between environmental risk conditions and health (i.e. place–health relations). This study in collaboration with the SOMMARTH DDESS geospatial platform aimed to assess corresponding field locations for:

1. Position accuracy of attributes within a geospatial village data layer, including location of the land parcel, shape and size;
2. Attribute accuracy of landuse classifications and road surface types assigned to the geospatial village data layer; and
3. Land parcel changes observed over a period of 2 years.

#### Study selection

**Fig. 2.**
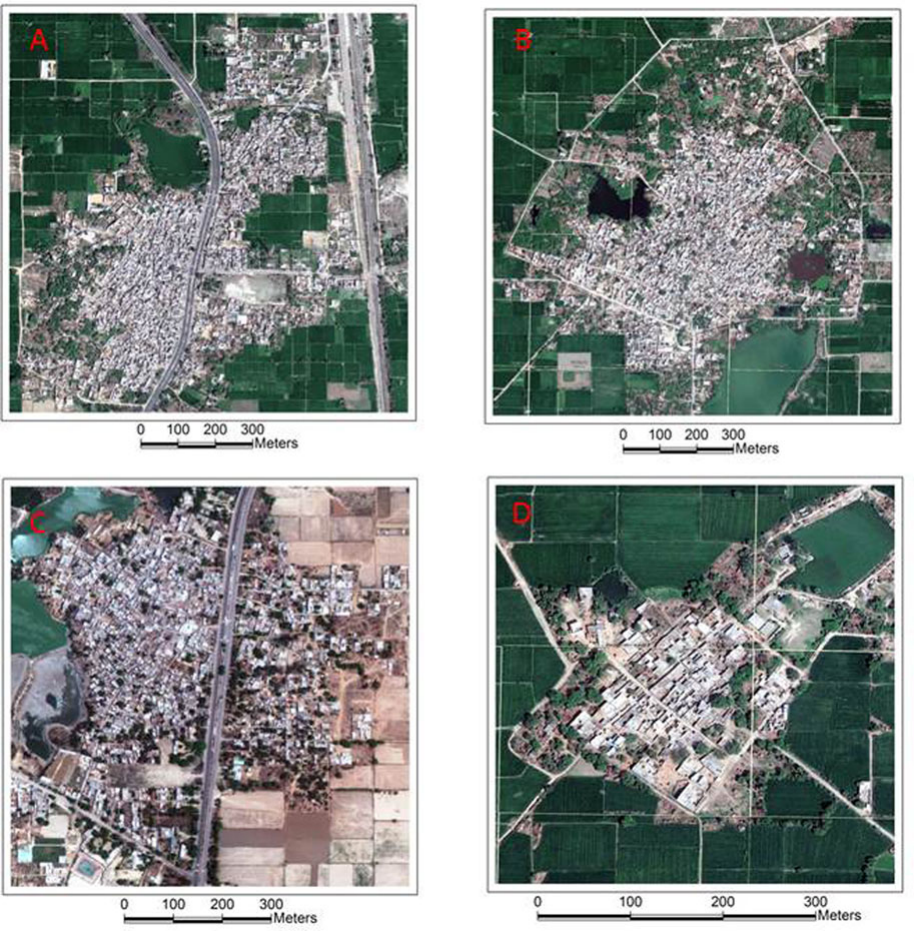
Sample frame circular and linear settlement patterns.

Villages already characterized within the GIS layer according to their built environment were eligible for selection, 32 of the 51 villages met this criteria. Eligible villages were assessed according to whether the village contained a *small* land parcel count (i.e. <1000 land parcels) or a *large* land parcel count (i.e. ≥1000 land parcels). Villages were assessed according to their settlement pattern as either *linear* or *circular*. A linear pattern of settlement has seen the development of dwellings and structures from a major highway, whereas a circular settlement begins in a central location radiating development in a circular pattern [24]. The final sampling frame, as indicated from satellite images in Fig. 2, included four groups for selection [(A) *small linear*, (B) *small circular*, (C) *large linear*, (D) *large circular*].

A total of six villages were selected to undertake an assessment, equating to 18.8% of characterized and 11.8% of the total study villages. Two villages were selected randomly from each of the *large linear* and *large circular* samples and one from each of *small linear* and *small circular.* The selected villages were additionally assessed according to the initial date of data collection. A final visual inspection of the selected villages occurred to ensure a diverse spatial coverage for the field exercise across the study region.

Prior to commencing the field exercise, a 5% sample of land parcels was identified within each selected village, with oversampling in villages with few land parcels to attain a minimum of 30 land parcels. A random selection of 5% of land parcels across the categories was undertaken, with under-sampling of ‘Residential’ (targeted 1% representation) to ensure a focus was maintained on a range of landuse types. Table 1 outlines the selected Palwal District villages also describing the population characteristics and proportions of residential, non-residential and mixed residential land parcels.

**Table 1.** Study area and sample characteristics

		Settlement pattern	Population			Land parcels				Sampled population and land parcels			
	Size		Total	Males	Females	Total	Residential	Non-residential	Mixed-residential	Sample	Residential	Non-residential	Mixed-residential
			*n*	*n* (%)	*n* (%)	*n*	*n* (%)	*n* (%)	*n* (%)	*n* (%)	*n* (%)	*n* (%)	*n* (%)
*Village*													
Bahin	Large	Circular	7369	3962 (53.8)	3407 (46.2)	1974	1181 (59.8)	724 (36.7)	69 (3.5)	102 (5.2)	24 (23.5)	59 (57.8)	19 (18.6)
Bamni Khera	Large	Linear	9720	5240 (53.9)	4480 (46.1)	2818	1643 (58.3)	107 (39.3)	68 (2.4)	123 (4.4)	36 (35.3)	75 (73.5)	12 (11.8)
Durgapur	Small	Linear	2384	1250 (52.4)	1134 (47.6)	658	421 (64.0)	218 (33.1)	19 (2.9)	34 (5.2)	8 (23.5)	22 (64.7)	4 (11.8)
Gahlab	Large	Circular	6363	3391 (53.3)	2972 (46.7)	1791	962 (53.7)	808 (45.1)	21 (1.2)	85 (4.7)	24 (28.2)	57 (67.1)	4 (4.7)
Garhi Vinoda	Small	Circular	570	299 (52.4)	271 (47.5)	183	91 (49.7)	88 (48.1)	4 (2.2)	40 (21.9)	23 (57.5)	17 (42.5)	0 (0.0)
Mitrol	Large	Linear	4760	2583 (54.3)	2177 (45.7)	1477	802 (54.3)	656 (44.4)	19 (1.3)	71 (4.8)	30 (55.6)	39 (54.9)	2 (3.7)
*Sample frame*													
	Large	Linear	14 480	7823 (54.0)	6657 (46.0)	4295	2445 (56.9)	763 (41.0)	87 (2.0)	194 (4.5)	66 (34.0)	114 (58.8)	14 (7.2)
	Large	Circular	13 732	7353 (53.4)	6379 (46.5)	3765	2143 (56.9)	532 (40.7)	90 (2.4)	187 (5.0)	48 (25.7)	116 (62.0)	23 (12.3)
	Small	Linear/circular	2954	1549 (52.4)	1405 (47.6)	841	512 (60.9)	306 (36.4)	23 (2.7)	74 (8.8)	31 (41.9)	39 (52.7)	4 (5.4)
Overall			31 166	16 725 (53.7)	14 441 (46.3)	8901	5100 (57.3)	601 (40.5)	200 (2.2)	455 (5.1)	145 (31.9)	269 (59.1)	41 (9.0)

Within the sampled villages, a total of 8901 land parcels had been characterized [median number of land parcels 1484; standard deviation (SD) 947.5]. Overall, 57.3% of land parcels represented a residential dwelling, with the lowest proportion (49.7) of residential within the small rural village of Garhi Vinoda, and the greatest proportion (64.0) of residential dwellings within Durgapur.

Table 1 also outlines a summary of sampled land parcels within each village, as well as summary statistics for each of the sampling frames and overall.

#### Field assessment

The sampled land parcels identified for assessment were highlighted on paper-based Sector-wise maps. Two assessors were involved, Assessor 1 (NH) was an independent place–health researcher and Assessor 2 (HRN) a trained geospatial researcher involved in the initial characterization. For this field assessment, a village check sheet, attribute table and marked paper-based Sector-wise map were utilized. A SOMAARTH DDESS village field worker was present during the assessment. These field workers were originally recruited from the local village and had knowledge of the region; moreover, for this study, it was desirable that the field worker was involved in the field-based participatory mapping. The SOMAARTH field team ensured that appropriate access was gained to the village, including notifying leaders (*Gram Panchayat*), and providing explanations to village members on the purpose of the field visit.

The two assessors oriented their direction within the village using physical features (e.g. rivers, water bodies) and major roads as landmarks to this positioning against a corresponding digitized paper-based map. Assistance was provided by a SOMAARTH field worker with input from local community members.

Using a defined check sheet of selected land parcels, the assessors systematically:

1. *Undertook* visual inspections of the sampled features and verified the presence or absence of the polygon feature on the digitized map.
2. *Recorded* whether the feature on the land parcel had been attributed (yes/no) and confirmed the location of this parcel along the road (e.g. fifth land parcel along road in an eastern direction).
3. *Engaged* with the village field worker, and if required assistance from a village member to reach consensus on the feature content.

Using the paper-based map as a field guide, position accuracy of the digitized land parcel was assessed for its relative size and shape using a step-out distance measure approach. The assessors used information associated with the land parcel (i.e. attribute table) to inform whether an environmental feature change had occurred since the participatory mapping exercise was undertaken. Photographs were taken of the features associated with the sampled land parcels and road surface confirming their presence and quality.

A post-field discussion was undertaken between the two assessors and field check sheets were entered into an Excel® (Microsoft, Washington, USA) spreadsheet and imported into an Access® (Microsoft) database for descriptive analyses, including proportion of missing attributes by village, and overall.

## Results

### Assessment of geospatial data accuracy

Table 2 highlights the descriptive results for position and attribute accuracy for each of the six sampled villages and summary by the sample frame (*small linear/circular*, *large linear*, *large circular*) and overall. Residential land parcels represented 31.9% of assessment sample, compared with 57.3% of land parcels represented in the study region, reflecting the under-sampling of residential locations to focus on adequate samples from a range of landuse types (e.g. commercial, industrial and agricultural).

**Table 2.** Position and attribute accuracy

	Sample	Attribute located		Attribute accuracy *proportion inaccurate*					
			Position accuracy *proportion inaccurate*	Building material type	Building classification	Participatory mapping landuse	Landuse classification		Road surface type
			Land parcel location, size and shape	*1. Pukka, 2. kutcha, 3. mixed*	*1. Residential, 2. non-residential, 3. mixed*	*Parcel landuse*	*Level II*	*Level III*	*1. Pukka, 2. kutcha, 3. kharanja*
	*N*	*n*	*n* (%)	*n* (%)	*n* (%)	*n* (%)	*n* (%)	*n* (%)	*n* (%)
*Village*									
Bahin	102	102	7 (6.9)	0 (0.0)	2 (2.0)	5 (4.9)	4 (3.9)	7 (6.9)	7 (6.9)
Bamni Khera	123	121	11 (9.1)	3 (2.5)	5 (4.1)	7 (5.8)	5 (4.1)	8 (6.6)	17 (14.0)
Durgapur	34	33	7 (21.2)	2 (6.1)	1 (3.0)	1 (3.0)	2 (6.1)	4(12.1)	0 (0.0)
Gahlab	85	85	2 (2.4)	2 (2.4)	5 (5.9)	7 (8.2)	6 (7.1)	7 (8.2)	10 (11.8)
Garhi Vinoda	40	40	1 (2.5)	0 (0.0)	0 (0.0)	1 (2.5)	1 (2.5)	1 (2.5)	0 (0.0)
Mitrol	71	71	2 (2.8)	0 (0.0)	0 (0.0)	0 (0.0)	1 (1.4)	2 (2.8)	14 (19.7)
*Sample frame*									
Large linear	194	192	13 (6.8)	3 (1.6)	5 (2.6)	7 (3.6)	6 (3.1)	10 (5.2)	31 (16.1)
Large circular	187	187	9 (4.8)	2 (1.1)	7 (3.7)	12 (6.4)	10 (5.3)	14 (7.5)	17 (9.1)
Small linear/circular	74	73	7 (9.6)	2 (2.7)	1 (1.4)	2 (2.7)	3 (4.1)	5 (6.8)	1(1.4)
Overall	455	452	29 (6.4)	7 (1.5)	13 (2.9)	21 (4.6)	19 (4.2)	29 (6.4)	49 (10.8)

Overall, 6.4% of land parcels were documented as having an error in the location, size or shape. The accuracy was least within a small village of Durgapur (21.2%), and there were no differences by sample frame. With respect to attribute accuracy, there were only 1.5% of land parcels that had an error in the building material type (metallic ‘*pukka*’, non-metallic ‘*kutcha*’ or mixed), and 2.9% relating to the building classification (as either residential/non-residential or mixed). During this exercise comparing existing geospatial data and participatory field observation with workers and village members, a 4.6% error in land parcel classifications was observed. The highest level of error was recorded within the sample frame ‘Large Circular’ (6.4%). Level II Landuse codes were misclassified for 4.2% of all land parcels. More prominent, however, was the misclassification observed for 6.4% (*n* = 29) of Level II Landuse codes.

The accuracy of the road surface type characterization ranged from complete accuracy (Durgapur and Garhi Vinoda) to the highest level of error observed within the village of Gahlab, with 10 incorrect classifications (11.8% of sampled parcels). The mean level of error for road surface type characterization was 8.2 (SD 6.85). Within the sample of parcels selected across the six villages, there were 10.8% (*n* = 49) that were incomplete.

In consultation with local community members, the researchers were able to determine any changes that had occurred within the villages since the time of the initial field survey (originally conducted 2011) and the Census taking place during 2013. Through this participatory approach, it was found that 17.4% of the land parcels had experienced a change in this 2-year period. Such results support the need to document the nature of land parcel changes due to rapid social and economic change within the region.

## Discussion

This paper outlined the processes of evaluation undertaken on a sample of geospatial data elements within a comprehensive public health surveillance platform representing rural Indian villages. The knowledge gained from this evaluation led to a process of system refinement for ongoing monitoring and surveillance of environmental risk conditions, as well as, the development of a verification tool to further integrate data elements into the geospatial-health platform. The research further suggests directions for improving geospatial-health data systems within low- and middle-income countries, as outlined below.

The results from this field-based observation of land parcel locations, according to size and shape, indicated the SOMAARTH DDESS had a high level of position accuracy. The study did not seek to assess the position accuracy of vector lines according to the gold standard satellite imagery. The ability to assess the precision of land parcels for the rural Indian village context would be highly unlikely. Furthermore, it was believed that any inaccuracy due to precision would not have an influence on the quality of the environmental indicators to be derived from the geospatial-health surveillance platform. The SOMAARTH DDESS architecture has been designed to capture, store and harmonize comprehensive datasets pertaining to the built environment. Furthermore, the platform has been intended for undertaking temporal spatial epidemiological analyses which require indicators expressed as counts or aggregated indices to assess variations in health and behavioural risk factors according to social, built and physical environmental features, such as weather and air quality, education, water and sanitation, and health care services.

The field-based observations provided an understanding into the attribute accuracy of the assigned landuse classifications and road surface types. The assessment of geospatial data quality has also identified the need for considering a more nuanced system for identifying road types and quality. Notably, there were observed misclassification of road types as either a lane or driveway (i.e. private ownership, presence of gate and/or door). Other examples included the consideration of road width (i.e. public alleyway or lane) and quality as these aspects impede accessibility within the village, particularly by car or motorbike. The appropriateness of landuse code assignment was assessed (i.e. logical consistency) and there was an identified need to consider the socio-spatial and cultural contexts in environmental data capture.

The contemporaneous capture of environmental features within the surveillance site is a crucial aspect to the overall geospatial data element accuracy, and thus, the utility for assessing social and built environmental features against health outcomes. Given the rapid social and economic changes being experienced within the surveillance site, the initial environmental data collection witnessed changes in land parcels prior to the demographic and health data collection. The verification tool ensured that data elements were contemporaneous for both environmental indicators and health outcomes. The need for surveillance systems and verification tools for spatial data accuracy are evident through undertaking an evaluation of SOMAARTH DDESS. Dixit *et al.* have demonstrated for this study context preliminary community-level findings on built and physical environmental exposures being associated with individual household socio-economic status [20]. The community and household-level exposures will be able to explain and quantify social determinants of health, as well as exploring associations with diverse individual health outcomes such as diabetes, cardiovascular and infectious disease [25]. Blakely and Woodward have outlined the importance of considering mismeasurement as a source of error affecting estimates of environmental exposures in relation to health outcomes [26]. Furthering discussion from Zhang *et al.* [16], the assessment of attribute accuracy (e.g. the ‘what’ of land parcel use and classification of road type) are additional threats to the validity of spatial epidemiological analyses, such as the planned analyses from longitudinal surveillance activities implemented in the Palwal District.

The evaluation exercise allowed for a process of reflection on cultural interpretations on these environmental constructs. As part of the baseline data collection, the participatory research approach included ‘ground truthing’ and interaction between village members, field workers and GIS technicians. Field observations highlighted the need to consider socio-spatial and cultural contexts (e.g. religious, cultural community infrastructure) within the coding framework for data elements. Subsequently, the landuse classification system was reviewed as part of a refinement exercise to be executed within all villages captured within the platform. The refinement resulted in the development of a verification tool that reflected the complexity of land parcel use and incorporation of a multi-level classification system.

A form indicating the options that a field worker may encounter during this exercise was detailed, including assessment of size, shape and location error. The informed procedure saw the type of change recorded according to the following classifications: (1) New dwelling/feature, (2) Under construction (on vacant land), (3) Demolished dwelling/feature, (4) Parcel/dwelling split and (5) Parcel dwelling merged. The time period in which this change had occurred was recorded as: (1) 1–3 months, (2) 3–6 months, (3) 6–12 months, (4) 1 or more years and (5) Not available/Don't know. The field worker provided open-ended comments and photographs to assist the GIS technician in update of the base village map.

A detailed rule book was developed to indicate examples relating to feature content (i.e. definition of cattle shade includes the need for the presence of shelter on the land parcel for the cattle). Training of field assessors and pilot testing was undertaken in June 2013, and the tool was refined accordingly before employment across all villages. The geospatial attribute verification tool (see Appendix file 1) was implemented across all 51 SOMAARTH DDESS villages between July and November 2013.

It is well-established that any population health research with priority populations must be driven by participatory approaches [27]. This resonates with prevailing perspectives in social geography which are also strongly influenced by participatory underpinnings [28]. GIS decision-making tools that incorporate local people's spatial knowledge have mainly been employed for development activities, local planning, resource management and community advocacy [29]. Such approaches to geospatial data collection do not privilege any one type of information but grant validity to all [30]; an approach that allows for both insider and outsider perspectives on spatial relationships within local communities (e.g. cultural notions of place). A mixed-method approach is also reflective of the reciprocal nature of the interaction of people within their local communities.

A strength of the study was its use of critical reflexivity, allowing for the processes of research and the information collected to be socially constructed [31]. The researcher employed insider and outsider perspectives to reflect on geospatial data accuracy within the spatial-health data surveillance system under development. The lead author (NH) was an ‘outsider’ both to the Indian culture and language, and villages that were being assessed. The second assessor (HRN) was involved for a period of 6 months in the technical application of the base maps, visiting around three villages, living within the greater region, and speaking Hindi. This approach to the research is intended to provide an enriched understanding of the social and cultural construction of the data system and aid in the processes of refinement [29].

An evaluation of the procedures to capture data indicated a high level of multi-disciplinary approaches integrated into the research, as reflected by the innovative nature to explore environmental risk conditions for cardiometabolic disease and its risk factors. There is however a crucial need to move from multi-disciplinary to trans-disciplinary perspectives in the assessment of transitions in health and lifestyle-related diseases. Such approaches include the integration of village members, field workers and desktop applications (i.e. geospatial technicians) to ensure digitization of environmental features was reflective of its position and content (i.e. attribute accuracy).

## Conclusions

Capturing change is complex, added to this complexity are both the people and the places in which they live, it occurs at different speeds and across different spatial geographies. The need to explore both levels of the system has been recognized and is being captured within a novel spatial-health data surveillance system within rural Indian villages. Regardless of the spatial nature of data, accuracy is a crucial aspect to population health information. The dynamic nature of a surveillance system needs to systematically deal with the inevitable change to environmental conditions, and the reciprocal influence on the people that reside within these local regions. Furthermore, the considerations of the contemporaneous nature of data for all levels of the system are vital to the future exploration of place and health relationships. This study has informed the refinement of data elements for all SOMAARTH DDESS Palwal study villages. The evaluation exercise contributes to our understandings on construction of public health surveillance systems within low- and middle-income countries. Furthermore, findings provide insights into considerations for assessing social, built and cultural environmental risk conditions in relation to health outcomes, such as lifestyle-related disease, which is burdening these local contexts as the rapid social, economic and landscape changes are experienced.

## Footnotes

† The notes appear after the main text.

## Note

1 ‘SOMAARTH’ is derived from Sanskrit: ‘Som’ meaning the highest form of physical, mental and spiritual health, and ‘Arth’ meaning money, wealth and resources. SOMAARTH envisions synergy between economic development, environment changes, social changes and health of the individual, family and community.
